# Spontaneous Hepatic Rupture Associated With Epstein-Barr Virus Negative Aggressive Natural Killer Cell Leukemia

**DOI:** 10.14740/wjon715w

**Published:** 2014-12-03

**Authors:** Nhu Thuy Can, Mei Lin Bissonnette, Muhammad Kamran Mirza, John Hart, Helen Te, Jane E. Churpek

**Affiliations:** a Department of Pathology, The University of Chicago, Chicago, IL, USA; b Department of Medicine, Section of Gastroenterology, The University of Chicago, Chicago, IL, USA; c Department of Medicine, Section of Hematology/Oncology, The University of Chicago, Chicago, IL, USA

**Keywords:** Natural killer cell, Leukemia, Hepatic rupture

## Abstract

Aggressive natural killer cell leukemia (ANKL) is a rare subtype of large granular lymphocyte (LGL) leukemia, which typically presents in young adults of Asian descent. It is an aggressive disease, characterized initially by fever, pancytopenia and hepatosplenomegaly, which rapidly progresses to organ failure and death over the course of months. Spontaneous hemorrhagic complications have been reported to occur in ANKL in a handful of case reports, including lethal intestinal and cerebral hemorrhage as well as splenic rupture. Here, we present a case of a 49-year-old man with Epstein-Barr virus (EBV)-negative ANKL who developed fatal spontaneous hepatic rupture approximately 4 months after initial diagnosis. To the best of our knowledge, this is first reported case of hepatic rupture associated with ANKL.

## Introduction

Aggressive natural killer cell leukemia (ANKL) is a rare subtype of large granular lymphocyte (LGL) leukemia that comprises < 0.1% of all hematologic neoplasms with only approximately 150 total cases reported in the literature [1-10]. ANKL typically presents in young adults (median, 42 years), particularly of Asian ethnicity [1, 3, 8, 10, 11] and has a highly aggressive clinical course characterized initially by fever, pancytopenia and hepatosplenomegaly, which quickly progresses to multi-organ failure, disseminated intravascular coagulation and death [1, 3, 4, 7, 10, 11]. Life-threatening hemorrhagic complications, including spontaneous splenic rupture thought to be secondary to angio-invasion by the neoplastic cells, have been reported [4, 11]. Here, we present a case of Epstein-Barr virus (EBV)-negative ANKL leading to hepatic capsular rupture and highlight the clinical challenges of this disorder. To the best of our knowledge, this is the first reported case of spontaneous hepatic rupture associated with ANKL [4, 11].

## Case Report

**Figure 1:**
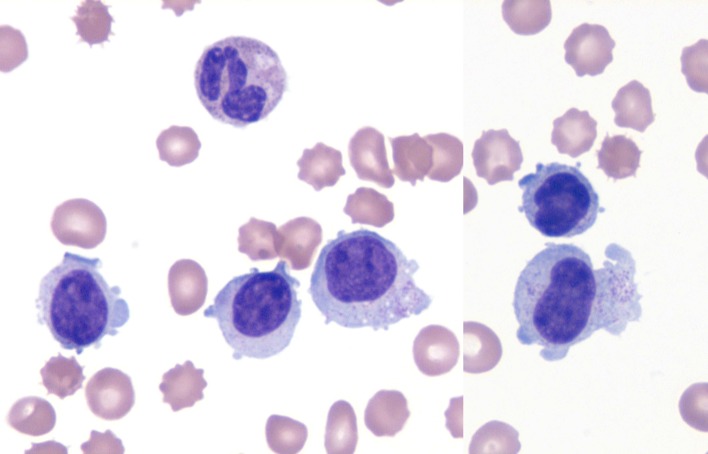
NK cell leukemia. Wright-Giemsa stain (× 1,000) of the peripheral smear. The specimen revealed an absolute lymphocytosis, comprised primarily of intermediate-sized lymphocytes with condensed chromatin and voluminous cytoplasm exhibiting large azurophylic granules (LGLs).

A 49-year-old man initially presented to an outside hospital 4 months prior with fever, night sweats and weight loss, along with mild splenomegaly and pancytopenia (WBC 2.3 × 10^3^/μL, hemoglobin 8.8 g/dL and platelets 47,000/μL). Lactate dehydrogenase (LDH), triglycerides and liver function tests were within normal limits. Review of the peripheral blood film revealed a population of large granular lymphocytes (Fig. 1). On flow cytometry, these accounted for 60-70% of all lymphocytes and demonstrated a natural killer (NK) cell phenotype: CD56+, CD57+, CD16+, granzyme B+, TIA-1+, CD3-, CD5-, CD2- with weak expression of CD8. A bone marrow biopsy exhibited a small population of NK cells with an identical phenotype. *In situ* hybridization for EBV-encoded RNA (EBER) was negative. Cytogenetic studies revealed a normal male karyotype. A pathologic diagnosis of chronic natural killer cell leukemia (CNKCL) was rendered; however, some clinical features, especially his debilitating daily fevers, night sweats and weight loss, were worrisome for ANKL. Initial treatment specific for CNKCL was attempted with oral cyclophosphamide and prednisone resulting in a partial response in blood counts only. Subsequent treatment with weekly pentostatin resulted in clinical worsening requiring hospital admission. At that point, multi-agent chemotherapy consisting of dexamethasone, methotrexate, ifosfamide, L-asparaginase and etoposide (SMILE regimen) was initiated for a clinical diagnosis of ANKL [5, 7, 8]. This treatment regimen led to the resolution of his B-symptoms, and he was discharged home.

On day 25 status post SMILE regimen and about 4 months since his initial diagnosis, he was readmitted with complaints of fever recurrence, diffuse abdominal pain, hypotension, nausea, vomiting and diarrhea. His physical examination was remarkable for pallor, icteric sclerae, decreased breath sounds at lung bases, mild and diffuse abdominal tenderness, and leg edema. Laboratory tests included: WBC 4.2 × 10^3^/μL (3.5 - 11 × 10^3^/μL), hemoglobin 7.1 g/dL (13.5 - 17.5 g/dL), platelet 54 × 10^3^/μL (150 - 450 × 10^3^/μL), serum albumin 2.8 (3.5 - 5.0 g/dL), alkaline phosphatase 355 IU/mL (30 - 120 IU/mL), aspartate aminotransferase 32 U/L (8 - 37 U/L), alanine aminotransferase 42 U/L (8 - 35 U/L), total bilirubin 3.8 mg/dL (0.1 - 1.0 mg/dL), conjugated bilirubin 2.0 mg/dL (0.1 - 1.0 mg/dL), LDH 237 U/L (116 - 245 U/L), fibrinogen 70 mg/dL (180 - 409 mg/dL) and prothrombin time (PT) 21.3 s (11.8 - 14.5 s). An infectious workup was negative.

The next hospital day, he developed localized right upper quadrant pain with rebound tenderness as well as a positive Murphy’s sign. An ultrasound of the liver revealed coarse, heterogenous echotexture of the liver, gallbladder wall with pericholecystic fluid, gallbladder sludge and adherent stones or polyps, suggestive of acute-on-chronic cholecystitis. A cholecystostomy tube was placed, resulting in hemodynamic stabilization, defervescence and clinical improvement. On the fourth hospital day, he developed clinical signs of peritonitis, necessitating an exploratory laparotomy. A perforated 2 mm duodenal ulcer was identified, and a Graham patch was performed. Intra-operatively, a liver biopsy of the left lobe was performed for further evaluation of the elevated liver chemistry tests, which demonstrated steatohepatitis, grade 3, stage 1, with superimposed cholestasis, raising the possibility of L-asparaginase toxicity. Although the sample was adequate in size, there were only rare portal tracts present in the specimen. Post-operatively, the patient continued to have fevers despite broad-spectrum antibiotics and negative blood cultures. On the seventh hospital day, the patient’s bilirubin had risen to 6 mg/dL despite an improvement in the serum alkaline phosphatase to 123 U/L. A cholecystostomy tube cholangiogram was performed to exclude tube obstruction, and a transjugular liver biopsy was concurrently obtained. The repeat liver biopsy was an adequate sample and demonstrated similar findings to the first biopsy. No NK cell infiltrates or hemophagocytic activity was appreciated. Almost 24 h later, the patient developed acute right upper quadrant abdominal pain and hemodynamic instability. His serum LDH had risen to 536 U/L, the WBC to 57.3 × 10^3^/μL from 5.4 × 10^3^/μL, and the hemoglobin decreased to 6.4 g/dL from 9.4 g/dL that morning. The WBC differential now demonstrated 89% neoplastic LGLs. He was emergently resuscitated and taken to the operating room for suspected intra-abdominal bleeding, where 8 L of blood was evacuated from the abdomen. Despite these efforts, he went into cardiac arrest and resuscitation was unsuccessful.

**Figure 2:**
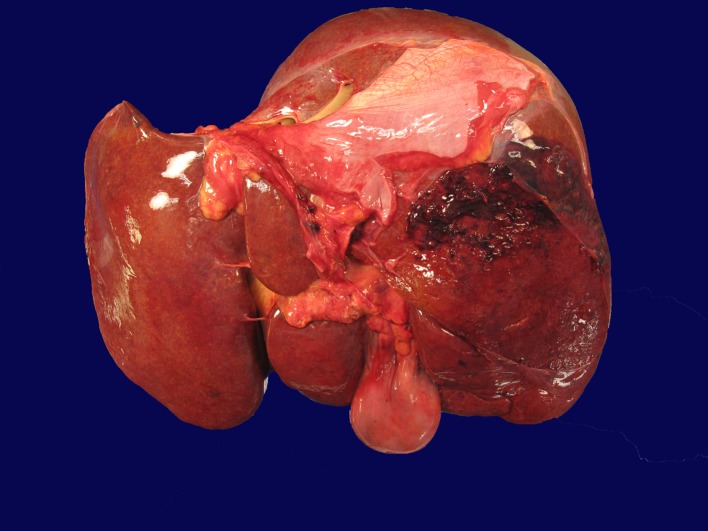
Liver with capsular and subcapsular parenchymal disruption on posterior surface of right lobe. This area was associated with an underlying intra-hepatic hematoma (6.5 × 5.5 × 5 cm).

Autopsy demonstrated an area of capsular and subcapsular parenchymal disruption (Fig. 2) with hemorrhage on the posterior surface of the right liver lobe with an associated peripherally located intra-hepatic hematoma (6.5 × 5.5 × 5 cm). Additionally, two intact cavernous hemangiomas (5 and 1.5 cm) were found in the right lobe of the liver and an intact biopsy site was identified on the surface of the left liver. The liver was examined extensively for a needle tract as evidence of hemorrhage from the transjugular biopsy, but none was found. Histologically, the hematoma and areas of hemorrhage contained numerous CD56+/CD7+ atypical lymphocytes, consistent with the patient’s NK cell leukemia.

The duodenal ulcer repair was in place and intact, but adjacent to this was an area of hemorrhage within the muscularis propria that contained CD 56+/CD7+ atypical lymphocytes. The patient’s NK cell leukemia also involved the vessels of the lung (including capillaries) and bone marrow. Additional pertinent autopsy findings included splenomegaly (639 g) and bilious pleural effusions (right 400 mL, left 300 mL). In summary, the cause of death was hemodynamic collapse from massive intra-abdominal bleeding originating from the liver.

## Discussion

ANKL is a rare hematologic neoplasm derived from neoplastic CD3-, CD56+ NK cells, which can range in morphologic appearance from LGLs to large, blast-like cells [1-12]. It is characterized by an acute presentation of fevers, jaundice, pancytopenia, and significant liver and spleen involvement, which often aggressively progresses to disseminated intravascular coagulation and multi-organ failure within weeks to months [1, 3, 4, 7, 10, 11]. ANKL is clinically challenging for several reasons. First, definitive clinical diagnosis may be quite difficult due to non-specific initial symptoms, the presence of limited numbers of neoplastic cells in the peripheral blood and/or bone marrow, and overlapping clinical features with a related disorder, CNKCL, which has an excellent prognosis [1, 3, 11, 13]. Second, treatment of ANKL is problematic because p-glycoprotein expressed by the neoplastic cells acts as an effluent pump for many chemotherapeutic drugs, resulting in limited response to most treatments [7, 8, 10]. Lastly, although median overall survival is < 2 months, a few patients treated with aggressive acute lymphoblastic leukemia-like multi-agent chemotherapy regimens and/or with autologous or allogeneic stem cell transplant in first remission have achieved survival of > 1 year [13, 14]. Thus, a rapid and accurate diagnosis of this entity is critical in order to prolong survival [1, 3, 4, 7, 8, 11].

In ANKL, the neoplastic cells are almost inevitably infected with EBV, but rare cases of EBV-negative ANKL have been reported with some appearing to have a better prognosis [1, 3-5, 7, 8, 10, 11]. Additionally, greater than half of ANKL cases have been associated with chromosomal abnormalities, most commonly a deletion in the region 6q21-q26 [1, 5]. Our patient’s clinical presentation and course were similar to those reported in the literature; however, EBER remained negative, and the patient demonstrated a normal male karyotype.

At autopsy, atypical LGLs were found to involve the bone marrow, lungs, duodenum and liver. Additionally, a large hematoma associated with capsular and subcapsular disruption was identified in the right liver lobe, consistent with hepatic capsular rupture that was the likely cause of hemodynamic compromise and death. We were not able to demonstrate ANKL involvement of the spleen due to extensive necrosis at the time of autopsy, but given the patient’s significant splenomegaly and diffuse organ involvement by ANKL, it is likely that neoplastic NK cells diffusely infiltrated the spleen.

Interestingly, spontaneous hemorrhagic complications have been reported to occur in ANKL. Okuno et al reported three cases of ANKL with lethal intestinal and cerebral hemorrhage, and Gao et al reported a case of ANKL presenting with splenic rupture [4, 11]. Our patient is the first reported case of spontaneous hepatic rupture in this disease. We considered several possible factors that may have contributed to the spontaneous hepatic rupture in our case. First, massive parenchymal infiltration by malignant cells in the liver and spleen may have led to capsule stretching, and ultimately, capsular disruption [3, 6, 11]. Second, our patient’s concomitant coagulopathy, as is commonly reported in this disease may have also been a contributing factor [4, 8, 11]. Additionally, the liver biopsy revealed features consistent with L-asparaginase toxicity, which may have also contributed to the development of coagulopathy. Finally, studies have shown that neoplastic NK cells are angio-invasive with the ability to cause massive necrosis through both vascular obstruction and diffuse infiltration of vessel walls [3, 4, 8]. Although our patient underwent two liver biopsies prior to the hepatic rupture, the first liver biopsy site (located in the opposite lobe) was intact, and the second biopsy was transjugular, distant from the peripherally located hematoma without an identifiable needle track, making the biopsies an unlikely source for subcapsular hemorrhage.

In conclusion, ANKL is an aggressive, often difficult to diagnose hematologic neoplasm that may be complicated by fatal hemorrhage. Patients with ANKL should be recognized as being at high risk for hemorrhagic complications and managed accordingly.
